# 4-Azido­methyl-7-methyl-2-oxo-2*H*-chromene-6-sulfonyl azide

**DOI:** 10.1107/S1600536810039693

**Published:** 2010-10-09

**Authors:** Mahantesha Basanagouda, Susanta K. Nayak, T. N. Guru Row, Manohar V. Kulkarni

**Affiliations:** a Department of Chemistry, Karnatak University, Dharwad 580 003, India; b Solid State and Structural Chemistry Unit, Indian Institute of Science, Bangalore 560 012, India

## Abstract

In the title compound, C_11_H_8_N_6_O_4_S, the plane of the coumarin aromatic ring is twisted by 17.2 (2)° with respect to the plane of the azide group bound to the methyl­ene substituent, whereas it is twisted by 83.2 (2)° to the plane of the azide attached to the sulfonyl group. The crystal structure is stabilized by weak C—H⋯O inter­actions, leading to the formation of dimers with *R*_2_^2^(12) graph-set motifs. These dimers are further linked by weak S—O⋯π and π–π contacts [centroid–centroid distance = 3.765 (2) Å], leading to the formation of a layered structure.

## Related literature

For azides, see: Scriven & Turnbull (1988 ▶); Amblard *et al.* (2009 ▶). For 4-azido­methyl­coumarin derivatives, see: Melavanki *et al.* (2008 ▶, 2009 ▶, 2010 ▶); Naik & Kullkarni (2010 ▶); Basanagouda *et al.* (2010 ▶). For hydrogen-bond motifs, see: Bernstein *et al.* (1995 ▶).
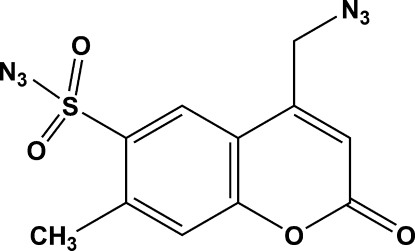


## Experimental

### 

#### Crystal data

C_11_H_8_N_6_O_4_S
*M* *_r_* = 320.29
Orthorhombic, 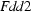

*a* = 13.5452 (12) Å
*b* = 27.952 (3) Å
*c* = 14.1107 (13) Å
*V* = 5342.5 (9) Å^3^
*Z* = 16
Mo *K*α radiation
μ = 0.27 mm^−1^
*T* = 292 K
0.24 × 0.16 × 0.10 mm

#### Data collection

Bruker SMART APEX CCD diffractometer
Absorption correction: multi-scan (*SADABS*; Sheldrick, 2008 ▶) *T*_min_ = 0.891, *T*_max_ = 0.973
11344 measured reflections
3124 independent reflections
2677 reflections with *I* > 2σ(*I*)
*R*_int_ = 0.025

#### Refinement

*R*[*F* ^2^ > 2σ(*F* ^2^)] = 0.041
*wR*(*F* ^2^) = 0.102
*S* = 1.05
3124 reflections
201 parameters
7 restraints
H-atom parameters constrained
Δρ_max_ = 0.26 e Å^−3^
Δρ_min_ = −0.21 e Å^−3^
Absolute structure: Flack (1983 ▶), 1438 Friedel pairs
Flack parameter: 0.09 (8)

### 

Data collection: *SMART* (Bruker, 2004 ▶); cell refinement: *SAINT* (Bruker, 2004 ▶); data reduction: *SAINT*; program(s) used to solve structure: *SHELXTL* (Sheldrick, 2008 ▶); program(s) used to refine structure: *SHELXL97* (Sheldrick, 2008 ▶); molecular graphics: *ORTEP-3 for Windows* (Farrugia, 1997 ▶) and *Mercury* (Macrae *et al.*, 2006 ▶); software used to prepare material for publication: *PLATON* (Spek, 2009 ▶).

## Supplementary Material

Crystal structure: contains datablocks global, I. DOI: 10.1107/S1600536810039693/dn2607sup1.cif

Structure factors: contains datablocks I. DOI: 10.1107/S1600536810039693/dn2607Isup2.hkl

Additional supplementary materials:  crystallographic information; 3D view; checkCIF report

## Figures and Tables

**Table 1:** Hydrogen-bond geometry (Å, °) *Cg* is the centroid of the C5–C10 ring.

*D*—H⋯*A*	*D*—H	H⋯*A*	*D*⋯*A*	*D*—H⋯*A*
C8—H8⋯O2^i^	0.93	2.52	3.416 (3)	161
S1—O4⋯*Cg*^ii^	1.42 (1)	2.96 (1)	3.931 (2)	128

Symmetry codes: (i) 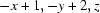; (ii) 
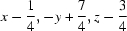
.

**Fig. 1.**
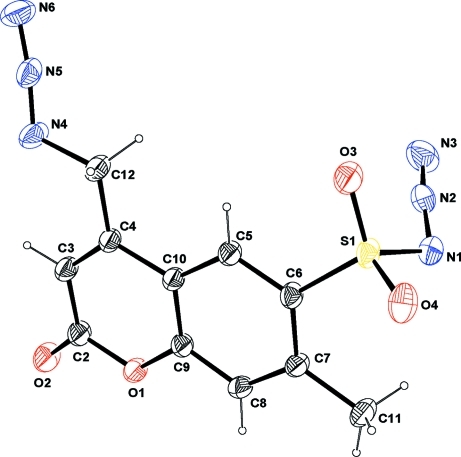
Molecular view of (I) with the atom labeling scheme. Ellipsoids are drawn at the 30% probability level. H atoms are represented as small spheres of arbitrary radii.

**Fig. 2.**
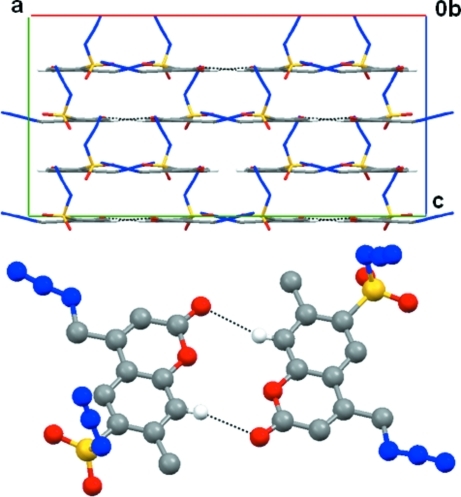
Molecular packing showing the layered structure stabilized by π–π and S═O···π contacts and the dimer formation through C—H···O intermolecular interactions. H atoms not involved in hydrogen bonding have been omitted for clarity

## supplementary crystallographic information

### Comment

The Chemisty of azides has been the subject of intensive investigations during the last 50 years because of its importance in preparative heterocyclic chemistry (Scriven *et al.*, 1988). The Cu(I)-catalyzed 1,3-dipolar cycloaddition reaction between alkynes and azides has been suitable for the synthesis of a large number of modified nucleosides, nucleotides and oligonucleotides with a broad range of applications (Amblard *et al.*, 2009). 4-Azidomethylcoumarin derivatives have aroused increasing interest because of their photophysical properties (Melavanki *et al.*, 2008, 2009, 2010; Naik *et al.*, 2010) and antimicrobial activities (Basanagouda *et al.*, 2010). As a part of our study on synthesis of biological active compounds, We report the crystal structure of 4-Azidomethyl-7-methyl-coumarin-6-sulfonyl azide.

The title compound (I), the molecular conformation (Fig.1) is preferred with the plane of the coumarin aromatic ring is 17.2 (2)° with respect to the plane of azide of methylene substituent whereas it is 83.2 (2)° to the plane of azide of sulfonyl group. The molecular arrangement is stabilized by the formation of dimer through C—H···O interactions with *R*_2_^2^(12) ring motif (Bernstein *et al.*, 1995)(Fig. 2) . Further, it is stabilized by S═O···π (Table 1, Cg being the centroid of the C5-C10 ring) and slippest π–π interaction (symmetry code (-1/4 + *x*, 7/4 - *y*, 1/4 + *z*) with centroid to centroid distance = 3.765 (2) Å, interplanar distance = 3.564 (1)Å and an offset angle of 18.8°, which form a layered structure (Fig. 2).

### Experimental

The 4–Bromomethyl–7–methyl–coumarin-6–sufonyl chloride (3.51 g, 0.01 mol) was taken in acetone (20 ml) in a round bottom flask. To this, solution of sodium azide(1.56 g, 0.024 mol) in water (6 ml) was added drop wise with stirring. The stirring was continued for 10 h. Then the reaction mixture was poured on to crushed ice (100 g). The separated solid was filtered and recrystallized from benzene to obtain a colorless solid in 62% yield, m.p. 132–133 °C; IR (KBr) cm^-1^ 1722 (C═O), 2130 (N_3_, azido); ^1^H NMR (CDCl_3_,300 MHz, TMS): δ 2.73 (s, 3H, C7—CH_3_), 4.62 (s, 2H, C4—CH_2_), 6.64 (s, 1H, C3—H), 7.31 (s, 1H, C8—H), 8.30 (s, 1H, C5—H); LC—MS: 321 (*M*+1). Anal. Calcd for C_11_H_8_N_6_O_4_S (%): Calcd. C, 41.25; H, 2.52; N, 26.24. Found C, 41.15; H, 2.46; N, 26.21.

### Refinement

All H atoms were fixed geometrically and treated as riding with C—H = 0.93Å (aromatic), 0.96Å (methyl) or 0.97Å (methylene) with U_iso_(H) = 1.2U_eq_(C) or U_iso_(H) = 1.5U_eq_(Cmethyl).

Rigid bond restraints on the ellipsoids were used for both azide groups.

### Crystal data

**Table:**

C_11_H_8_N_6_O_4_S	*D*_x_ = 1.593 Mg m^−^^3^
*M**_r_* = 320.29	Melting point: 406 K
Orthorhombic, *F**d**d*2	Mo *K*α radiation, λ = 0.71073 Å
Hall symbol: F 2 -2d	Cell parameters from 400 reflections
*a* = 13.5452 (12) Å	θ = 1.0–28.0°
*b* = 27.952 (3) Å	µ = 0.27 mm^−^^1^
*c* = 14.1107 (13) Å	*T* = 292 K
*V* = 5342.5 (9) Å^3^	Hexagonal, pale yellow
*Z* = 16	0.24 × 0.16 × 0.10 mm
*F*(000) = 2624	

### Data collection

**Table:**

Bruker SMART APEX CCD diffractometer	3124 independent reflections
Radiation source: fine-focus sealed tube	2677 reflections with *I* > 2σ(*I*)
graphite	*R*_int_ = 0.025
φ and ω scans	θ_max_ = 28.0°, θ_min_ = 2.2°
Absorption correction: multi-scan (*SADABS*; Sheldrick, 2008)	*h* = −17→0
*T*_min_ = 0.891, *T*_max_ = 0.973	*k* = −36→0
11344 measured reflections	*l* = −18→18

### Refinement

**Table:**

Refinement on *F*^2^	Secondary atom site location: difference Fourier map
Least-squares matrix: full	Hydrogen site location: inferred from neighbouring sites
*R*[*F*^2^ > 2σ(*F*^2^)] = 0.041	H-atom parameters constrained
*wR*(*F*^2^) = 0.102	*w* = 1/[σ^2^(*F*_o_^2^) + (0.0532*P*)^2^ + 3.5426*P*] where *P* = (*F*_o_^2^ + 2*F*_c_^2^)/3
*S* = 1.05	(Δ/σ)_max_ = 0.001
3124 reflections	Δρ_max_ = 0.26 e Å^−^^3^
201 parameters	Δρ_min_ = −0.21 e Å^−^^3^
7 restraints	Absolute structure: Flack (1983), 1438 Friedel pairs
Primary atom site location: structure-invariant direct methods	Flack parameter: 0.09 (8)

### Special details

**Table:**

Geometry. All e.s.d.'s (except the e.s.d. in the dihedral angle between two l.s. planes) are estimated using the full covariance matrix. The cell e.s.d.'s are taken into account individually in the estimation of e.s.d.'s in distances, angles and torsion angles; correlations between e.s.d.'s in cell parameters are only used when they are defined by crystal symmetry. An approximate (isotropic) treatment of cell e.s.d.'s is used for estimating e.s.d.'s involving l.s. planes.
Refinement. Refinement of *F*^2^ against ALL reflections. The weighted *R*-factor *wR* and goodness of fit *S* are based on *F*^2^, conventional *R*-factors *R* are based on *F*, with *F* set to zero for negative *F*^2^. The threshold expression of *F*^2^ > σ(*F*^2^) is used only for calculating *R*-factors(gt) *etc*. and is not relevant to the choice of reflections for refinement. *R*-factors based on *F*^2^ are statistically about twice as large as those based on *F*, and *R*- factors based on ALL data will be even larger.

### Fractional atomic coordinates and isotropic or equivalent isotropic displacement parameters (Å^2^)

**Table:**

	*x*	*y*	*z*	*U*_iso_*/*U*_eq_	
S1	0.85568 (4)	0.83758 (2)	0.50177 (5)	0.04317 (17)	
O1	0.47627 (11)	0.93758 (5)	0.47959 (14)	0.0462 (4)	
O2	0.31442 (12)	0.93929 (6)	0.49151 (18)	0.0610 (6)	
O3	0.83522 (13)	0.78772 (6)	0.5081 (2)	0.0663 (6)	
O4	0.92870 (14)	0.85453 (7)	0.43818 (16)	0.0598 (5)	
N1	0.89734 (14)	0.85727 (8)	0.60674 (18)	0.0507 (5)	
N2	0.86115 (15)	0.83563 (8)	0.67677 (19)	0.0511 (6)	
N3	0.8356 (2)	0.81892 (11)	0.7443 (3)	0.0727 (8)	
N4	0.37159 (16)	0.76434 (8)	0.4783 (2)	0.0643 (8)	
N5	0.36677 (14)	0.72360 (8)	0.4993 (2)	0.0494 (5)	
N6	0.35192 (18)	0.68477 (9)	0.5172 (3)	0.0715 (8)	
C2	0.38675 (17)	0.91435 (8)	0.4834 (2)	0.0439 (6)	
C3	0.38818 (17)	0.86301 (8)	0.4776 (2)	0.0436 (6)	
H3	0.3284	0.8467	0.4752	0.052*	
C4	0.47186 (17)	0.83767 (7)	0.47560 (17)	0.0370 (5)	
C5	0.65857 (15)	0.84097 (8)	0.48129 (17)	0.0372 (5)	
H5	0.6634	0.8078	0.4815	0.045*	
C6	0.74334 (15)	0.86865 (8)	0.48503 (18)	0.0368 (5)	
C7	0.74063 (16)	0.91904 (8)	0.48102 (17)	0.0366 (5)	
C8	0.64872 (16)	0.94005 (8)	0.47701 (18)	0.0397 (6)	
H8	0.6439	0.9732	0.4741	0.048*	
C9	0.56344 (16)	0.91278 (8)	0.47726 (18)	0.0371 (5)	
C10	0.56574 (15)	0.86284 (7)	0.47717 (17)	0.0354 (5)	
C11	0.83110 (18)	0.95033 (9)	0.4850 (2)	0.0507 (7)	
H11A	0.8697	0.9457	0.4287	0.076*	
H11B	0.8698	0.9420	0.5395	0.076*	
H11C	0.8114	0.9833	0.4893	0.076*	
C12	0.47394 (17)	0.78398 (8)	0.4712 (2)	0.0482 (7)	
H12A	0.5036	0.7738	0.4119	0.058*	
H12B	0.5138	0.7716	0.5227	0.058*	

### Atomic displacement parameters (Å^2^)

**Table:**

	*U*^11^	*U*^22^	*U*^33^	*U*^12^	*U*^13^	*U*^23^
S1	0.0256 (2)	0.0409 (3)	0.0630 (4)	0.0027 (2)	−0.0001 (3)	−0.0076 (3)
O1	0.0301 (7)	0.0290 (7)	0.0796 (13)	0.0036 (6)	−0.0053 (9)	0.0013 (8)
O2	0.0330 (8)	0.0437 (9)	0.1064 (17)	0.0092 (7)	−0.0043 (12)	−0.0052 (11)
O3	0.0391 (9)	0.0387 (9)	0.1212 (18)	0.0081 (7)	−0.0105 (13)	−0.0100 (12)
O4	0.0346 (9)	0.0800 (14)	0.0647 (12)	0.0016 (9)	0.0090 (9)	−0.0099 (11)
N1	0.0365 (11)	0.0561 (13)	0.0595 (14)	−0.0065 (9)	−0.0045 (10)	0.0077 (11)
N2	0.0321 (11)	0.0519 (14)	0.0693 (16)	0.0087 (10)	−0.0030 (11)	0.0056 (12)
N3	0.0538 (14)	0.0844 (19)	0.080 (2)	0.0118 (13)	0.0103 (15)	0.0282 (17)
N4	0.0326 (10)	0.0369 (11)	0.123 (3)	−0.0022 (8)	−0.0061 (13)	0.0063 (13)
N5	0.0336 (9)	0.0501 (13)	0.0645 (14)	−0.0067 (8)	−0.0001 (11)	−0.0062 (13)
N6	0.0595 (15)	0.0435 (13)	0.111 (3)	−0.0128 (11)	0.0026 (15)	0.0127 (15)
C2	0.0319 (11)	0.0366 (12)	0.0631 (17)	0.0037 (8)	−0.0045 (11)	−0.0015 (12)
C3	0.0293 (10)	0.0351 (11)	0.0662 (17)	−0.0035 (8)	−0.0032 (11)	0.0006 (11)
C4	0.0292 (10)	0.0308 (10)	0.0511 (14)	−0.0019 (8)	−0.0037 (10)	−0.0017 (11)
C5	0.0299 (10)	0.0315 (10)	0.0502 (15)	0.0013 (8)	−0.0011 (10)	−0.0032 (10)
C6	0.0271 (9)	0.0356 (10)	0.0476 (13)	0.0018 (8)	−0.0006 (10)	−0.0048 (10)
C7	0.0346 (11)	0.0318 (10)	0.0433 (14)	−0.0053 (8)	0.0007 (10)	−0.0012 (10)
C8	0.0382 (11)	0.0271 (10)	0.0540 (15)	−0.0019 (8)	−0.0027 (12)	0.0018 (10)
C9	0.0293 (11)	0.0326 (11)	0.0495 (13)	0.0025 (8)	−0.0036 (10)	0.0000 (10)
C10	0.0284 (10)	0.0306 (10)	0.0473 (13)	−0.0019 (8)	−0.0023 (10)	−0.0003 (9)
C11	0.0356 (11)	0.0391 (12)	0.077 (2)	−0.0089 (9)	0.0015 (14)	−0.0030 (14)
C12	0.0310 (11)	0.0321 (10)	0.0814 (19)	−0.0031 (9)	−0.0035 (12)	−0.0037 (12)

### Geometric parameters (Å, °)

**Table:**

S1—O4	1.417 (2)	C4—C12	1.502 (3)
S1—O3	1.4237 (18)	C5—C6	1.386 (3)
S1—N1	1.678 (3)	C5—C10	1.399 (3)
S1—C6	1.768 (2)	C5—H5	0.9300
O1—C9	1.370 (3)	C6—C7	1.410 (3)
O1—C2	1.376 (3)	C7—C8	1.378 (3)
O2—C2	1.208 (3)	C7—C11	1.507 (3)
N1—N2	1.258 (3)	C8—C9	1.384 (3)
N2—N3	1.116 (4)	C8—H8	0.9300
N4—N5	1.178 (3)	C9—C10	1.396 (3)
N4—C12	1.495 (3)	C11—H11A	0.9600
N5—N6	1.133 (3)	C11—H11B	0.9600
C2—C3	1.438 (3)	C11—H11C	0.9600
C3—C4	1.337 (3)	C12—H12A	0.9700
C3—H3	0.9300	C12—H12B	0.9700
C4—C10	1.453 (3)		
			
O4—S1—O3	120.17 (13)	C7—C6—S1	121.23 (16)
O4—S1—N1	102.39 (12)	C8—C7—C6	116.80 (19)
O3—S1—N1	109.36 (14)	C8—C7—C11	119.3 (2)
O4—S1—C6	110.61 (12)	C6—C7—C11	123.9 (2)
O3—S1—C6	108.77 (11)	C7—C8—C9	121.3 (2)
N1—S1—C6	104.26 (11)	C7—C8—H8	119.4
C9—O1—C2	121.45 (17)	C9—C8—H8	119.4
N2—N1—S1	113.88 (19)	O1—C9—C8	116.16 (18)
N3—N2—N1	173.0 (3)	O1—C9—C10	121.68 (19)
N5—N4—C12	115.0 (2)	C8—C9—C10	122.1 (2)
N6—N5—N4	172.8 (3)	C9—C10—C5	117.18 (19)
O2—C2—O1	116.5 (2)	C9—C10—C4	117.67 (19)
O2—C2—C3	126.3 (2)	C5—C10—C4	125.13 (19)
O1—C2—C3	117.18 (19)	C7—C11—H11A	109.5
C4—C3—C2	122.8 (2)	C7—C11—H11B	109.5
C4—C3—H3	118.6	H11A—C11—H11B	109.5
C2—C3—H3	118.6	C7—C11—H11C	109.5
C3—C4—C10	119.02 (19)	H11A—C11—H11C	109.5
C3—C4—C12	123.1 (2)	H11B—C11—H11C	109.5
C10—C4—C12	117.89 (19)	N4—C12—C4	110.29 (18)
C6—C5—C10	120.1 (2)	N4—C12—H12A	109.6
C6—C5—H5	119.9	C4—C12—H12A	109.6
C10—C5—H5	119.9	N4—C12—H12B	109.6
C5—C6—C7	122.3 (2)	C4—C12—H12B	109.6
C5—C6—S1	116.36 (17)	H12A—C12—H12B	108.1
			
O4—S1—N1—N2	−160.77 (19)	S1—C6—C7—C11	3.5 (4)
O3—S1—N1—N2	−32.3 (2)	C6—C7—C8—C9	0.2 (4)
C6—S1—N1—N2	83.9 (2)	C11—C7—C8—C9	−177.3 (2)
C9—O1—C2—O2	175.3 (2)	C2—O1—C9—C8	−177.7 (2)
C9—O1—C2—C3	−4.6 (4)	C2—O1—C9—C10	1.0 (4)
O2—C2—C3—C4	−175.0 (3)	C7—C8—C9—O1	175.7 (2)
O1—C2—C3—C4	4.8 (4)	C7—C8—C9—C10	−3.0 (4)
C2—C3—C4—C10	−1.4 (4)	O1—C9—C10—C5	−175.7 (2)
C2—C3—C4—C12	179.0 (3)	C8—C9—C10—C5	2.9 (3)
C10—C5—C6—C7	−2.6 (4)	O1—C9—C10—C4	2.5 (3)
C10—C5—C6—S1	174.00 (18)	C8—C9—C10—C4	−178.8 (2)
O4—S1—C6—C5	134.2 (2)	C6—C5—C10—C9	−0.1 (3)
O3—S1—C6—C5	0.2 (3)	C6—C5—C10—C4	−178.3 (2)
N1—S1—C6—C5	−116.4 (2)	C3—C4—C10—C9	−2.3 (4)
O4—S1—C6—C7	−49.1 (2)	C12—C4—C10—C9	177.4 (2)
O3—S1—C6—C7	176.8 (2)	C3—C4—C10—C5	175.8 (2)
N1—S1—C6—C7	60.3 (2)	C12—C4—C10—C5	−4.5 (4)
C5—C6—C7—C8	2.6 (4)	N5—N4—C12—C4	−161.8 (3)
S1—C6—C7—C8	−173.89 (19)	C3—C4—C12—N4	−5.1 (4)
C5—C6—C7—C11	179.9 (3)	C10—C4—C12—N4	175.3 (2)

### Hydrogen-bond geometry (Å, °)

**Table:**

Cg is the centroid of the C5–C10 ring.

**Table:**

*D*—H···*A*	*D*—H	H···*A*	*D*···*A*	*D*—H···*A*
C8—H8···O2^i^	0.93	2.52	3.416 (3)	161
S1—O4···Cg^ii^	1.417 (2)	2.963 (2)	3.931 (2)	128

Symmetry codes: (i) −*x*+1, −*y*+2, *z*; (ii) *x*−1/4, −*y*+7/4, *z*−3/4.
